# Modernizing CTSA hub evaluation: an integrated system for performance monitoring and translational science impact assessment

**DOI:** 10.3389/frma.2025.1534394

**Published:** 2025-03-31

**Authors:** Maryam Gholami, Elizabeth Johnson, Kera Swanson, Carlos Rojas, Daniel Bouland

**Affiliations:** ^1^Altman Clinical and Translational Research Institute (ACTRI), University of California San Diego Health, La Jolla, CA, United States; ^2^The San Diego Center for AIDS Research (CFAR), University of California San Diego Health, La Jolla, CA, United States

**Keywords:** impact assessment, progress tracking, process monitoring, evaluation, translational science benefits model

## Abstract

**Background:**

The Evaluation Unit at the Altman Clinical and Translational Research Institute (ACTRI) implemented a balanced scorecard model in conjunction with a project management tool to consolidate data collection for progress monitoring, strategic alignment, and impact assessment. This approach aims to streamline communication and enhance information accessibility for all partners. We developed an efficient system for collecting, analyzing, and reporting key information on unit progress, impact, and alignment with institutional goals. The Translational Science Benefits Model (TSBM) was proposed as a framework to evaluate the broader impact of our translational research, beyond immediate scientific advancements, across clinical, societal, economic, and policy domains.

**Methods:**

The ACTRI Evaluation Unit initially adapted the balanced scorecard (BSC) to the research environment, substituting business perspectives with research grant aims. In its second iteration, the BSC was integrated into Monday.com, a project management platform, to create customized, real-time monitoring dashboards for each unit within the institute. The Evaluation Unit's 3.0 version further adapted the TSBM to assess the broader impacts of unit activities. Quarterly data collection was implemented, and partners were trained in impact assessment and dashboard usage. This process began in early 2023 and is ongoing.

**Results:**

Eleven monitoring dashboards were developed and successfully implemented across the institute. The new system facilitated more efficient data collection and reporting, reducing communication overhead and increasing the frequency of updates. The data collected were utilized to draft annual reports as well as inform strategic planning and executive sessions.

**Conclusions:**

Integrating the TSBM into our existing BSC framework, combined with a project management tool, effectively streamlined impact assessment and progress monitoring. This approach not only enhanced data collection and reporting efficiency but also encouraged units to align their goals and activities with desired impacts, thereby strengthening the institute's overall strategic focus.

## Introduction

In the rapidly evolving landscape of clinical and translational science, effective evaluation, impact assessment, and strategic alignment have become increasingly crucial for research institutions (Trochim et al., 2013). The University of California San Diego (UCSD) Altman Clinical and Translational Research Institute (ACTRI) has recognized this need and implemented an innovative approach to address these challenges.

The Evaluation Unit at the ACTRI plays a pivotal role in supporting program and unit leaders in planning, executing, and monitoring their activities. This support ensures that all efforts are aligned with organizational goals and contribute to continuous performance improvement. To facilitate this process, the Evaluation Unit identified the need for a robust measurement system that would enable the ACTRI units to assess their progress and develop targeted improvement plans (Croucher et al., 2018; Himanen and Puuska, 2022).

In 2012, building upon the success of the Balanced Scorecard (BSC) implementation in the academic department of medicine (Bouland et al., 2011), the ACTRI's Evaluation Unit adopted an electronic version of the BSC for strategic management purposes (Hoyo and Bouland, 2022).

The Balanced Scorecard (BSC) functions as a strategic management framework that enables organizations to translate their vision into measurable objectives (Kaplan and Norton, 1992).

Rather than focusing exclusively on financial metrics, the BSC provides a multidimensional approach to performance assessment. The framework facilitates the conversion of strategic goals into actionable initiatives with clear performance indicators. By monitoring progress across multiple perspectives—financial performance, customer relationships, internal processes, and organizational learning—leadership teams gain comprehensive visibility into organizational effectiveness. This integrated approach to performance measurement allows executives to develop a holistic understanding of operations and make data-driven decisions that support long-term strategic objectives. The BSC thus bridges the gap between strategic planning and operational execution, ensuring alignment throughout the organization.

In 2022, to further enhance efficiency and streamline progress management, the Evaluation Unit migrated the BSC to Monday.com, a widely used project management platform (Monday.com, n.d.). It was noted that customization of the BSC required more costly programming resources. Monday.com offered a visual and collaborative workspace where teams could create and customize workflows, manage tasks, track projects, and collaborate in real-time. This migration provided a more flexible and adaptive platform to meet the unique needs of the ACTRI's various units and workflows.

Complementing this approach, the ACTRI has incorporated the Translational Science Benefits Model (TSBM) to evaluate the broader impact of translational research beyond immediate scientific advancements (Luke et al., 2018). The Translational Science Benefits Model (TSBM) provides a structured framework for understanding and measuring how scientific research creates tangible value beyond academia. This model maps the progression of knowledge from laboratory discoveries to real-world applications across four distinct domains:

Clinical benefits: Improvements in patient care, treatment protocols, diagnostic capabilities, and health outcomes resulting from research implementation in healthcare settings.

Community benefits: Enhanced public health practices, increased health literacy, improved access to care, and strengthened community partnerships that collectively improve population health.

Economic benefits: Financial returns including cost savings, efficiency gains, new commercial opportunities, job creation, and broader economic development stemming from scientific advances.

Policy benefits: Evidence-based changes to regulations, guidelines, standards, and public policies that improve systems and structures affecting health and wellbeing.

The TSBM helps researchers, funders, and stakeholders systematically identify, track, and communicate the diverse impacts of their work. By providing a comprehensive evaluation framework that extends beyond traditional academic metrics (like publications and citations), the TSBM enables more accurate assessment of research's societal value and helps justify continued investment in scientific enterprise.

By integrating the BSC model with Monday.com and the TSBM framework, the ACTRI has developed a comprehensive system for data collection, progress monitoring, strategic alignment, and impact assessment. This integrated approach was developed with the goal of serving multiple purposes:

1. Streamline communication processes across the organization.
2. Enhance information accessibility to units, operations, and center leaders.
3. Provide a robust framework for evaluating the broader impact of translational research.
4. Allow for real-time tracking and management of projects and tasks.
5. Facilitate the continuous quality improvement plans based on data-driven insights.

This paper will describe the implementation of this unified system at the ACTRI, evaluation plans for its effectiveness in streamlining processes, strengthening strategic alignment, and providing a more comprehensive assessment of research impact. We will discuss the challenges encountered, the solutions developed, and the potential implications of this approach for other research institutions seeking to improve their evaluation and management processes.

## Methods

In version 1.0, the process began with the ACTRI Evaluation Unit adapting the traditional BSC to suit the research environment. Instead of using standard business perspectives, we substituted these with research grant aims, aligning the scorecard more closely with our institutional goals (Bouland et al., 2011; Hoyo and Bouland, 2022). This initial adaptation laid the groundwork for the subsequent iterations of our evaluation system.

The BSC provided a more robust tool for linking management to strategy. Our primary objective was to create a centralized hub offering a comprehensive solution for all partners involved in our research activities (Kaufmann and Kock, 2022; Santos et al., 2022).

In the second phase, version 2.0, we integrated the adapted BSC into Monday.com, a versatile project management platform, where multiple individuals can have access to each dashboard to import and export information. This incorporation allowed us to create customized, real-time monitoring dashboards for each unit within the institute (Monday.com, n.d.). The synergy between the BSC framework and Monday.com's functionality provided a robust foundation for our evaluation and monitoring efforts.

The widespread adoption and success of Monday.com within the ACTRI led to a strategic decision to integrate the BSCs with this platform. Monday.com was familiar to users for project management efforts, time tracking functions, process flows, and as a general go-to place for file storage and communication consolidation.

We designed the Monday.com dashboards with the specific goal of capturing only essential information to meet the needs of our hubs strategic planning goals. Our main objective is to use this data effectively for various purposes. Box 1 outlines the purposes for which we collect information through the dashboards.

Box 1 **Streamlining multiple processes using the information collected by the ACTRI Evaluation Unit.**

***RPPR tables and narratives:*** These dashboards serve as a valuable resource for populating NIH Research Performance Progress Report (RPPR) tables and crafting accompanying narratives.

***Grant proposals:*** The information collected is instrumental in formulating comprehensive grant proposals.

***Annual external advisory committee meeting presentations:*** We rely on the data to create compelling presentations for our annual external advisory committee meetings.

***Internal executive committee meetings:*** The dashboards facilitate productive discussions and decision-making during internal executive committee meetings.

***Administrative and financial analyses and management:*** We utilize the data for in-depth administrative and financial analyses, aiding effective management practices.

Building on this foundation, the third version (3.0) of our evaluation system further incorporated the Translational Science Benefits Model (TSBM; Luke et al., 2018). This addition enabled a more comprehensive assessment of the broader impacts of unit activities across various domains, enhancing our ability to capture and communicate the full value of our research outcomes (Miovsky et al., 2023; Sperling et al., 2023). This integration creates a powerful system that not only tracks operational metrics but also captures the multidimensional societal value of our research initiatives. By implementing this framework through the accessible Monday.com platform, we've created an intuitive, centralized solution that supports strategic decision-making while reducing administrative burden. This approach enables ACTRI to demonstrate accountability to stakeholders, optimize resource allocation, and ultimately accelerate the translation of scientific discoveries into meaningful benefits across clinical, community, economic, and policy domains. Box 2 details specific information that we collect in the centralized dashboards.

Box 2 **Information collected by ACTRI Evaluation using the integrated system.**

***Specific aims:*** Clear and concise articulation of the unit's specific objectives and goals.

***Metrics for each aim:*** Quantifiable measures or key performance indicators (KPIs) that allow for objective assessment of progress toward the specified aims.

***Strategies for achieving the aims:*** Detailed actionable plans outlining the steps and methods to be employed in order to attain the stated aims.

***Connect boards:*** Linking project-specific Monday dashboards to the relevant units' BSC dashboard provides easier access to a comprehensive view of each project.

***Alignment with overall ACTRI goals:*** Demonstrating how these aims and strategies contribute to our overarching ACTRI goals.

***Impact assessment based on TSBM:*** Evaluation of the anticipated or observed impacts of the aims and strategies in alignment with the TSBM.

***Additional columns (added based on feedback):*** These columns were introduced based on suggestions from unit leaders and project managers, addressing specific needs and enhancing the utility of the platform.

### Implementing Monday balanced scorecards

To ensure the effectiveness of the integrated system, we implemented a quarterly data collection schedule. The quarterly data collection schedule serves as the operational backbone of our streamlined evaluation system, establishing a consistent rhythm for information gathering and analysis across the institution. This structured approach ensures that leadership has access to current metrics, enabling responsive management decisions based on the latest available data. Our training program complements this schedule, focusing on building capacity among partners to not only utilize the Monday.com interface but also to develop critical analytical skills for meaningful impact assessment.

We provided one-on-one training sessions in conjunction with the implementation of the new system. The dual focus of our training—impact evaluation methodology and technical platform proficiency—has proven essential for successful deployment. By equipping unit leaders with both conceptual understanding and practical skills, we've fostered organizational buy-in and created a sustainable culture of evidence-based decision making. The guidelines outlined in Box 3 provide a replicable framework that addresses common implementation challenges. This comprehensive approach to system adoption has been instrumental in transforming our evaluation framework from a theoretical model into an embedded institutional practice that drives continuous improvement and demonstrates the full value of our translational research efforts.

Box 3 **Implementation strategies to integrate the BSC-Monday.com at the ACTRI.**

***Board introduction:*** Initially, we created customized dashboards for all 11 units across ACTRI, populating them with specific aims, strategies, and metrics in alignment with the grant proposal.

***Guided orientation:*** We then conducted comprehensive walkthroughs with unit leaders and project managers, elucidating the purpose behind the dashboards and providing a detailed explanation of each column's role. This included clarifying what needed to be added to the dashboards.

***TSBM training:*** We provided translational science benefits models training for all unit leaders and project managers and shared learning materials with them.

***Open dialogue:*** We actively engaged in discussions, welcomed questions, and actively sought feedback regarding the complexity and feasibility of the task. This collaborative approach led to the inclusion of additional columns, based on valuable input from unit leaders and project managers.

***Continuous support:*** Throughout the dashboard completion process, we offered ongoing support and maintained open lines of communication.

***Deadline setting:*** We established deadlines, allowing a 2-week timeframe for the initial phase of tasks. Subsequently, we revisited the dashboards, collaborating with unit leaders and project managers to ensure all missing elements were addressed.

***Strategic communication:*** We leveraged upcoming milestones, such as the annual RPPR, grant proposal preparations, and the impending EAC meeting, as opportunities to communicate the importance of completing the dashboards. This strategic approach ensured that the dashboards would serve as invaluable resources for these critical events.

The resulting integrated platform serves multiple functions, creating a versatile tool for our institution. Unit leaders and project managers can directly input data into the system, ensuring real-time updates on project progress and outcomes. The Executive Committee can monitor unit progress through customized dashboards and reports. Furthermore, the system facilitates the extraction of information for various purposes, including National Institute of Health (NIH) Research Performance Process Reports (RPPRs), grant applications, Executive Advisory Committee (EAC) presentations, and various internal applications and reports (Trochim et al., 2013).

## Results

Prior to implementing our integrated system, our evaluation process faced significant operational challenges. Data collection relied heavily on email communications, requiring multiple follow-up messages to unit leaders to gather necessary information. Responses were often unstructured, inconsistent, and frequently delayed. The annual Research Performance Progress Report (RPPR) preparation was particularly problematic, characterized by last-minute data gathering, incomplete information, and a rushed compilation process due to the absence of systematic tracking throughout the year. This reactive approach led to potential omissions and increased stress on both unit leaders and evaluation staff.

Implementing our integrated BSC and Monday.com system, along with the TSBM, led to significant improvements in the institute's monitoring and evaluation processes. The outcomes of this implementation fall into three domains for ongoing quality improvement: system deployment, operational efficiency, and strategic impact.

### System deployment

We successfully developed 11 monitoring dashboards across the institute. These dashboards were tailored to the specific needs and functions of different units within our organization, ensuring comprehensive coverage of all key areas of our operations. The widespread adoption of these dashboards demonstrates the scalability and adaptability of our combined approach to diverse research contexts within the institute. One common challenge that we experienced across most units was initial skepticism that was overcome with the ease of use.

### Operational efficiency

The new system led to marked improvements in operational efficiency, particularly in the areas of data collection and reporting:

1. Data Collection: The integration of Monday.com with our adapted BSC framework streamlined the data collection process. Unit leaders and project managers were able to input data directly into the system, leading to more timely and accurate information gathering.
2. Reporting Efficiency: The centralized nature of the system significantly reduced the time and effort required to compile and generate reports. This efficiency gain was particularly notable in the preparation of annual reports, where the readily available, well-organized data expedited the drafting process.
3. Communication Overhead: We observed a substantial reduction in communication overhead. The real-time nature of the Monday.com platform, combined with the structured data input, minimized the need for frequent follow-ups and clarifications.

### Strategic impact

The application of this new evaluation system shows several promising impacts on our strategic planning and decision-making processes.

The following examples are in the process to be realized:

1. Informed Strategic Planning: We will use the information gathered for the 2025 strategic planning retreat. The system's comprehensive and up-to-date data provided by will provide leaders with a valuable resource for making informed decisions, grounded in accurate insights on the performance and progress of various units.
2. Executive Committee: The system proves to be an invaluable tool during Executive Committee sessions. The ability to access real-time data and generate on-the-spot reports enhances the quality and depth of discussions, leading to more informed decision-making at the highest levels of institute leadership. We are currently scheduled for periodic presentations at Executive Committee meetings and plan to use the system as our data source.

The following impacts of our integrated system have been realized:

3. Holistic Impact Assessment: The incorporation of the TSBM into our monitoring system allowed for a more comprehensive assessment of our research activities. This broader perspective on impact helped align our strategic goals with the wider benefits of our translational science efforts.
4. Adaptive Management: The regular influx of data and the ease of generating reports allowed for more adaptive management practices. Leaders were able to identify trends, challenges, and opportunities more quickly, enabling timely adjustments to strategies and resource allocations.

While initial observations suggest improvements in operational efficiency and strategic capabilities, we plan to rigorously evaluate the full impact and effectiveness of our integrated approach through a comprehensive evaluation plan (see Table 1). This systematic assessment will help quantify the system's contribution to data-driven decision-making and impact assessment across the institute, providing evidence-based insights into its value and identifying areas for optimization.

**Table 1:** Evaluation plan for the system designed to track progress and impact at the ACTRI.

**Evaluation component**	**Metrics/methods**	**Data collection method**
**Data quality**		
Completeness	Percentage of fields completed	Automated audit
Timeliness	On-time submission rate	System timestamps
Accuracy	Error rate in reported data	Random verification
**User experience**		
System usability	User satisfaction scores	User surveys
Training effectiveness	Training completion rates	Training records
User engagement	Dashboard access frequency	Usage logs
**Operational efficiency**		
Report generation	Time saved in report creation	Time tracking
Communication efficiency	Reduction in data-related emails	Email analytics
RPPR preparation	Time spent on RPPR compilation	Time tracking
**Strategic impact**		
Decision making	Use of data in strategic decisions	Strategic review
Executive discussions	Presentations in Executive meetings	Meeting minutes
Cross-unit Collaboration	Number of collaborative projects	Project tracking

### Evaluation plan

Our evaluation of the amalgamated BSC, Monday.com, and TSBM system will employ a mixed-methods approach to assess both the implementation process and outcomes. This comprehensive evaluation framework (Table 1) will help ensure continuous improvement and maximize the system's value for all partners.

## Discussion

The implementation of our BSC and Monday.com system, integrating the TSBM, marks a significant advancement in the ACTRI's evaluation and management practices. Preliminary results indicate promising improvements in operational efficiency, data accessibility, and strategic decision-making capabilities. These outcomes support the broader goals of the Clinical and Translational Science Award (CTSA) program to improve the efficiency and impact of translational research (Center for Advancing Translational Sciences Institutes of Health, 2025).

The creation of this centralized system has streamlined our evaluation and reporting processes, improved data accessibility, and enhanced our ability to assess and communicate the broader impacts of our research activities. Initiated in early 2023, this ongoing process of integration, data collection, and training continues to evolve to meet the changing needs of our institution and the broader landscape of clinical and translational science.

Our approach builds on previous efforts within the CTSA consortium to create robust evaluation frameworks (Rubio, 2013; Selker, 2020). While the Common Metrics system did not employ the same technological platform, it shared our goal of fostering a more responsive, data-driven research environment. Our assimilation of the BSC with Monday.com offers a novel solution to challenges identified in earlier CTSA evaluation efforts, particularly enhancing real-time data accessibility and maximizing the value of invested efforts (Rubio, 2013; Welch et al., 2021).

The incorporation of the TSBM into our evaluation framework is particularly noteworthy. This model, developed by Luke et al. (2018), provides a structured approach to assessing the broader impacts of translational science across clinical, community, economic, and policy domains. By incorporating the TSBM with our BSC and project management tool, we've created a system that not only tracks operational metrics but also captures the wider societal benefits of our research. This aligns with the growing emphasis within the CTSA program on demonstrating the real-world impact of translational science (Ruiz et al., 2022).

Our success in implementing this system across 11 diverse units within our institute demonstrates its scalability and adaptability. The reduction in communication overhead and increased frequency of updates observed in our results address a common challenge faced by CTSA hubs: the need for timely and accurate data to inform decision-making (Ruiz et al., 2022). Our system's ability to facilitate more efficient data collection and reporting is particularly valuable in the context of the annual RPPR required by the National Center for Advancing Translational Sciences (NCATS).

The potential strategic impact of our system, particularly its role in informing executive sessions and strategic planning, aligns with the CTSA program's emphasis on data-driven leadership (Center for Advancing Translational Sciences Institutes of Health, 2025). By providing real-time, comprehensive data on both operational performance and broader impacts, our system enables a more agile and responsive approach to managing translational science initiatives. This capability is increasingly important as CTSA hubs are called upon to demonstrate their value and adapt to changing research priorities (Center for Advancing Translational Sciences Institutes of Health, 2025).

### Limitations

While our preliminary results are promising, it's important to acknowledge some limitations. The implementation of such a comprehensive system requires significant investment in terms of time, resources, and organizational change management. Future research could explore the cost-effectiveness of this approach compared to other evaluation strategies employed across the CTSA consortium. Additionally, longitudinal studies will be necessary to fully assess the long-term impact and effectiveness of this system on strategic management, research outcomes, and translational efficiency.

### Future directions

To further enhance our understanding of ACTRI's broader contributions to building capacity in translational science, we plan to expand the evaluation system in future iterations. Upcoming versions will incorporate additional metrics and domains that reflect evolving priorities of translational science. For instance, we will assess workforce development impacts, including researcher training, mentorship outcomes, and diversity within research teams. Additionally, we plan to evaluate the effectiveness of community partnerships by tracking engagement levels, collaborative outcomes, and the impact of community-driven research.

## Conclusion

Our integrated approach to evaluation and management represents a significant step forward in addressing the complex challenges faced by CTSA hubs. By combining established frameworks like the BSC and TSBM with modern project management tools like Monday.com, we've created a system that enhances both operational efficiency and strategic capabilities. As the CTSA program continues to evolve, such innovative approaches to evaluation and management will be crucial in maximizing the impact of translational science investments.

## Data Availability

The original contributions presented in the study are included in the article/supplementary material, further inquiries can be directed to the corresponding author.
